# Immunological fingerprint of 4CMenB recombinant antigens via protein microarray reveals key immunosignatures correlating with bactericidal activity

**DOI:** 10.1038/s41467-020-18791-0

**Published:** 2020-10-05

**Authors:** E. Bartolini, E. Borgogni, M. Bruttini, A. Muzzi, M. Giuliani, S. Iozzi, R. Petracca, M. Martinelli, S. Bonacci, S. Marchi, C. Brettoni, C. Donati, G. Torricelli, S. Guidotti, M. Domina, C. Beninati, G. Teti, F. Felici, R. Rappuoli, F. Castellino, G. Del Giudice, V. Masignani, M. Pizza, D. Maione

**Affiliations:** 1 GSK, Siena, Italy; 2 University of Siena, Siena, Italy; 3 Unit of Computational Biology, Research and Innovation Centre, Fondazione Edmund Mach, San Michele all’Adige, Italy; 4 University of Messina, Messina, Italy; 5 University of Molise, Campobasso, Italy

**Keywords:** Antibodies, Protein vaccines, Bacterial infection

## Abstract

Serogroup B meningococcus (MenB) is a leading cause of meningitis and sepsis across the world and vaccination is the most effective way to protect against this disease. 4CMenB is a multi-component vaccine against MenB, which is now licensed for use in subjects >2 months of age in several countries. In this study, we describe the development and use of an ad hoc protein microarray to study the immune response induced by the three major 4CMenB antigenic components (fHbp, NHBA and NadA) in individual sera from vaccinated infants, adolescents and adults. The resulting 4CMenB protein antigen fingerprinting allowed the identification of specific human antibody repertoire correlating with the bactericidal response elicited in each subject. This work represents an example of epitope mapping of the immune response induced by a multicomponent vaccine in different age groups with the identification of protective signatures. It shows the high flexibility of this microarray based methodology in terms of high-throughput information and minimal volume of biological samples needed.

## Abstract (web summary)

4CMenB is an approved multi-component vaccine against Serogroup B meningococcus. Here the authors develop a protein microarray for three major 4CMenB antigenic components (fHbp, NHBA and NadA) and describe antibody repertoires in sera from vaccinated infants, adolescents and adults correlating with bactericidal response.

## Introduction

The development of effective vaccines against *Neisseria meningitidis* of serogroup B (MenB) has been challenging due to the poor immunogenicity of its capsular polysaccharide and to the variability of the major outer membrane proteins. The identification of sub-capsular antigens, more conserved in sequence and able to induce bactericidal antibodies, has been possible through the reverse vaccinology approach^1,2^. This has led to the development and registration of the multicomponent, recombinant, 4CMenB vaccine, containing two fusion proteins, Neisserial heparin-binding antigen-GNA1030 (NHBA-GNA1030) and GNA2091-factor H-binding protein (GNA2091-fHbp), plus the recombinant *N. meningitidis* adhesin A (NadA), in combination with the detergent-extracted outer membrane vesicles (OMV) derived from the epidemic meningococcal NZ98/254 strain^3,4^. Bactericidal activity, that is the ability of the antibodies to kill bacteria in presence of complement, is the established correlate of protection for MenB^5,6^. However, little is known about the nature of the protein epitopes or antigenic domains inducing bactericidal antibodies in different age groups. This knowledge is important because different epitopes can show different degree of immunogenicity depending on age^7^. Therefore, improved vaccine efficacy could be achieved with a deeper understanding of the kinetics of antibody immunity from infancy to adulthood^8^.

Various techniques, such as X-ray crystallography^9,10^, nuclear magnetic resonance (NMR) spectroscopy^11,12^, and hydrogen–deuterium exchange mass spectrometry (HDX-MS)^13,14^ have been applied successfully to map antibody-recognised epitopes. Although these approaches are extremely informative, they are generally time and sample consuming and require highly specific purified monoclonal antibodies, as they are not easily applicable to the epitope mapping of polyclonal sera. Other methods include the phage display technology described firstly by Smith^15^, that has been used for decades to investigate protein–protein interactions including antigen–antibody recognition^16–18^. An alternative approach is based on peptide libraries, i.e. arrays of overlapping synthetic peptides encompassing the entire primary structure of the target antigen^19^. These peptides are generally of only 12–15 aa in length, therefore present mainly linear epitopes whereas they are not representative of more complex epitopes made by residues located on discontinuous regions. Nevertheless, the use of peptide libraries has been successfully applied to the identification of protein domains or epitopes involved in binding to antibodies generated by vaccination^20,21^ or autoantibodies^22–24^.

Nowadays, a faster and more powerful technology to perform epitope mapping is represented by protein microarrays, which allow analysing simultaneously short and long peptides that are representative of all immunogenic regions of an antigen, including both linear and conformational epitopes, with the further advantage of using only minimal volumes of biological samples. Proteomic microarrays generated by spotting full-length antigens are largely used to profile responses to bacterial infections^25,26^ or following vaccination^27–29^ or as diagnostic tool^30^.

Differently, only few studies have attempted at characterising the antibody repertoires in vaccinees based on antigenic fingerprinting^31^ or at analysing the pattern of epitopes that are preferentially targeted by antibodies in subjects of different age.

In the present work, we first used phage display libraries to characterise the antibody response in different age populations, highlighting an age-dependent recognition profile. The results obtained by phage display, combined with an in silico design of potential epitopes, allowed to develop a proteomic platform to measure the diversity of antibody binding profiles for different MenB vaccine antigens and to get a more precise picture on individual heterogeneity of the antibody response to 4CMenB. In particular, we designed, generated and validated a microarray platform suitable to detect antibodies raised against conformational and linear epitopes of the three main antigens included in 4CMenB, i.e. NadA, NHBA and fHbp. These protein microarrays were used to analyse individual sera from subjects of different ages before and after vaccination with 4CMenB. The defined immunosignatures were then correlated with sero-protection.

## Results

### Antigen recognition profiles revealed by phage display

To identify the immunogenic regions of 4CMenB antigens recognised by antibodies elicited upon vaccination, and to evaluate whether the antibodies induced in subjects of different ages are directed against different epitopes, we screened three antigen-specific phage display libraries expressing peptides of the three major 4CMenB antigens (fHbp, NadA and NHBA) with pools of human sera from infants, adolescents and adults immunised with 4CMenB. In particular the adult pool was composed by 29 sera, the adolescent pool contained 12 sera and the pool of infant sera included 40 samples, all of them collected 1 month after last vaccination dose. The three libraries were constructed by randomly cloning fragments of each vaccine component, ranging from approximately 66–333 amino acid (aa), in the bacteriophage lambda vector λKM4^17,32^) to generate N-terminal fusion proteins with gpD, a lambda-phage capsid component that is exposed on phage surface. The average size of the recombinant inserts encoded fragments was 110 aa for GNA2091-fHbp, 123 aa for NadA and 136 aa for NHBA-GNA1030. Affinity selections of the three phage display libraries with pool of sera from vaccinees were performed to identify immunoreactive phages whose DNA inserts were then sequenced.

**Fig. 1:**
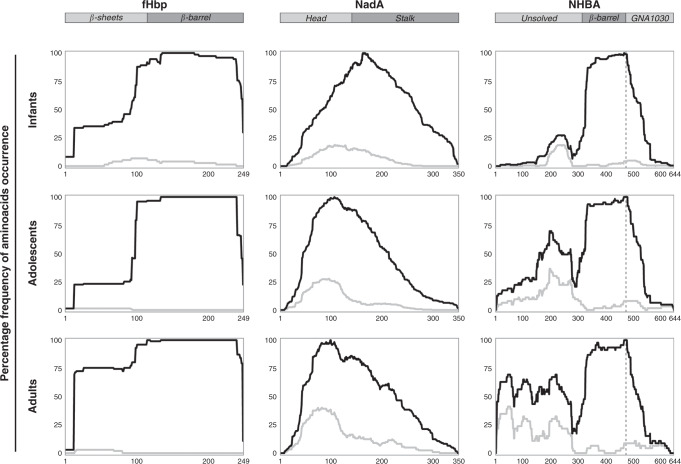
Phage display selection frequency of aminoacids along antigen primary structures. Sera were analysed as pool by age group (40 infant, 12 adolescent and 29 adult samples). Aminoacid position in the sequence of reference proteins from N-term to C-term is reported on horizontal axis, while vertical axis shows the relative frequency of occurrence for the corresponding aminoacid in the in-frame affinity selected fragments. Frequency refers to the total in-frame fragments analysed in each experiment. Light grey curves are produced by considering only short fragments spanning less than 100 residues.

In 4CMenB the fHbp antigen is fused to the accessory protein GNA2091 to enhance its immunogenicity. fHbp is able to bind the human factor H (hfH) and is composed of a relatively conserved N-terminal taco-shaped β-barrel and a variable C-terminal eight-stranded β-barrel domain; crystal structure of the fHbp:hfH complex has revealed that the binding of human fH engages both fHbp protein domains^33^. The immunoscreening of the GNA2091-fHbp library was conducted including a competition step with unbound GNA2091 to maximise the antibody recognition of the fHbp component. For the adult and adolescent groups, an additional affinity selection step was used to obtain a comparable number of in-frame clones among the three age groups. Pooled sera from the three different groups of subjects reacted very similarly with only long fragments comprising either the full-length molecule or the whole C-terminal β-barrel domain of fHbp that were selected. This was particularly evident for adult sera (Fig. 1).

NadA is a trimeric protein and is structurally composed of an extended trimeric coiled-coil stalk domain and a distal N-terminal head domain characterised by the presence of short wing-like structures^34,35^. Following one round of affinity selection on the NadA library, pooled sera from all three groups showed a good recognition along the entire protein, with longer selected fragments (more than 100 aa) covering the entire protein sequence and smaller fragments (less than 100 aa) mainly mapping on the first 150 residues corresponding to the globular head domain^34,35^. These finding suggest the presence of both linear and conformational epitopes in the globular head, whereas the stalk domain seems to include mainly conformational epitopes.

Interestingly, there was an age-dependent increase in the frequency of short fragments mapping on the N-terminal part of the protein (Fig. 1).

The NHBA antigen is present in 4CMenB as a fusion protein with the accessory protein GNA1030 to further increase its immunogenicity^4^. The N-terminus of NHBA is predicted as an intrinsically unfolded region, while the structure of NHBA C-terminal region has been solved by NMR, revealing an eight-stranded β-barrel domain^36^. The NHBA protein is able to bind heparin in vitro through an Arg-rich region located in a flexible loop between the β-barrel of the C-terminus and the unsolved N-terminus; this region is the target of two proteases, the meningococcal NalP and human lactoferrin^36^. The antibody-selected fragments from the NHBA-GNA1030 library showed that the antibody response was mainly directed towards the C-terminal region of NHBA in all age groups. The reactivity on just large fragments containing the entire C-terminal β-barrel domain suggested that the antibodies are mainly directed against conformational epitopes. In contrast, smaller fragments of less than 100 residues were mapped on the NHBA N-terminal domain, which is predicted to be mainly unfolded^37^. Moreover, while infant and adolescent sera showed an antibody response directed against a small region of the NHBA N-terminus, corresponding to residues within G150 and K270, the antibody response in adult sera was directed against the entire N-terminal domain, spanning from residue P4 to K270 (Fig. 1).

### Protein array as accurate tool for epitope mapping analysis

While the phage display approach was instrumental in the identification of antigenic regions recognised by pooled sera, the protocol described above was not suitable for the analysis of the antibody response of individual subjects. To this aim, we employed protein arrays encompassing the entire length of the three MenB antigens.

The 53 more represented immunoreactive fragments identified by phage display were selected to build a widely representative protein microarray with additional 36 antigen fragments containing potentially relevant epitopes from each of the three main vaccine components on the basis of secondary structure computational analysis. We designed these additional fragments with different lengths and in a such way that the epitopes were represented in more than one single fragment, to increase the chances that all potential epitopes were correctly represented.

The 4CMenB antigen array was then generated by spotting 106 different recombinant purified proteins on nitrocellulose-coated glass slides. To verify that spotted protein fragments are properly spotted on the microarrays, a panel of sixwell characterised monoclonal antibodies targeting conformational or linear epitopes on each of the three main 4CMenB antigen components was used: 30G4 and 12C1 anti-fHbp^13,14^; 33E8, 6E3 and 9F11 anti-NadA^35,38^ and 31E10 anti-NHBA^39^. All fragments including the pre-identified mAbs-specific epitopes were found reactive, as expected (Supplementary Fig. 1), confirming that protein fragments which sequence preserve the tertiary structure maintain their folding when spotted on the array.

Moreover, these microarrays have been previously used in Giuliani et al.^40^ to map the epitopes recognised by a large panel of human monoclonal antibodies (HumAbs) elicited by 4CMenB vaccination. In Giuliani et al. a panel of HumAbs was also characterised by additional approaches, such as Pep-Scan, HDX-MS and the epitope mapping results showed a high concordance with the data generated by the protein array, further supporting the reliability of this technology^40^.

MenB protein arrays were then probed with 221 individual sera. Since that not all of the sera tested as pool in phage display were still available, we randomly selected sera from the same clinical trials, increasing the numbers of subjects to analyse. In total, 30 sera from adults, 46 sera from adolescents and 69 sera from infants were tested. Pre-immune (PI) sera from adolescents and adults were used as a control (PI sera from infants were not available). Sera reactivity was evaluated by detecting total IgG bound to each protein spot using fluorescently labelled anti-human IgG antibodies and measuring the mean fluorescence intensity (MFI) values for each protein fragment. 23,426 data points (221 sera × 106 spotted proteins) were produced in 221 hybridisation experiments.

The datasets generated by Protein microarray experiments and analysed during the current study are available in the Gene Expression Omnibus database GEO GSE152785.

**Fig. 2:**
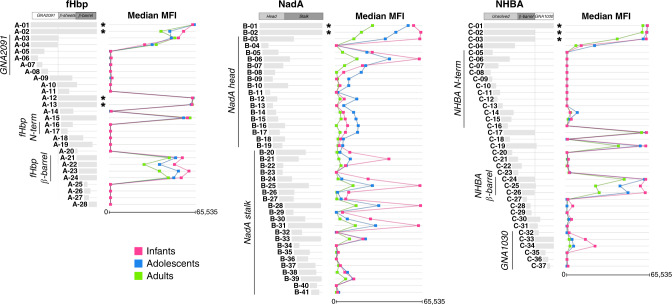
Protein microarray analysis unveils the reactivity profile of 4CMenB antigens. Each horizontal light grey bar represents a single protein or protein fragment spotted in the microarray for GNA2091-fHbp (A-01 to A-28), NadA (B-01 to B-41) and NHBA-GNA1030 (C-01 to C-37). The corresponding coloured squares on their right show the median antibody binding reactivity against that fragment in all tested sera from human vaccines, grouped by age: green squares for adult subject sera, blue squares for sera from adolescents, and pink squares for sera from infants. Stars indicate the full-length or whole fusion forms of each antigen.

In summary, PI sera were consistently not reactive, whereas most of the sera reacted with full-length antigens in all their different forms upon vaccination (Fig. 2, star labelled fragments). Despite some variability among subjects, the median MFI values of full-length fHbp and NHBA were approximately 60,000 (close to upper limit of detection of 65,535) without any statistically significant difference between age groups. On the other hand, adult sera (median MFI: 26,704; IQR: 29,991) showed a significantly reduced reactivity against full-length NadA (Wilcoxon rank-sum: *p* < 0.0001) if compared to both adolescent (median MFI: 53,014; IQR: 14,969) and infant sera (median MFI: 57,018; IQR: 4631).

In Fig. 2, left panel, we observed that the antibody response was mostly directed towards the eight-stranded C-terminal β-barrel domain of fHbp in all subjects irrespective of their age. The different full-length forms of fHbp (fragments A-12, A-13) and the accessory protein GNA2091 (fragments A-03, A-04), as well as the entire fusion protein (fragments A-01, A-02) were abundantly recognised by most of the sera. Fragments not comprising the entire C-terminal β-barrel were generally not recognised, suggesting the prevalence of antibodies directed against conformational epitopes. Infant sera recognised the β-barrel-containing fragments (A-21 to A-24) with a greater intensity value compares to sera from adolescents and even more compared to adult sera (Wilcoxon rank-sum: INF > ADO (*p* = 1.5e−06); ADO > ADU (*p* = 0.0004); INF > ADU (*p* = 7.7e−13)).

The extended protein chip analysis revealed that the most reactive fragments of NadA were located along the whole protein sequence, in either the N-terminal head or the C-terminal stalk domains (Fig. 2, central panel). Adult sera showed low reactivity to most of the NadA specific fragments as compared to the other age groups. Conversely, median MFI values reached in infant sera were greater than those obtained from adolescents and adults in nearly all fragments, especially the longer ones either in the N-terminal head (B-03 and B-06) or C-terminal stalk (B-25, B-28 and B-31). Interestingly, adolescents exhibited the most intense recognition of shorter fragments (B-11 to B-19) located in the head portion of NadA (Wilcoxon rank-sum: ADO > INF (*p* = 3.8e−11); INF > ADU (*p* = 2.6e−08); ADO > ADU (*p* < 2.2e−16)).

Finally, most of the sera reacted with the C-terminal β-barrel domain of the NHBA-GNA1030 (C-24, C-25 and C-26), while the recognition of the central portion of the protein (fragments C-14 and C-15) was highly variable and only in adolescent sera the C-14 fragment reached a positive, although low, median MFI value of 8200 (IQR: 26,256) (Fig. 2, right panel). Those fragments containing only the Arg-rich region (C-21, C-22 and C-23) were not recognised by most of the sera and only infants’ sera recognised the accessory protein GNA1030, though exclusively as full-length protein (Fig. 2, right panel).

### Cluster analysis showed individual vaccine immunosignatures

**Fig. 3:**
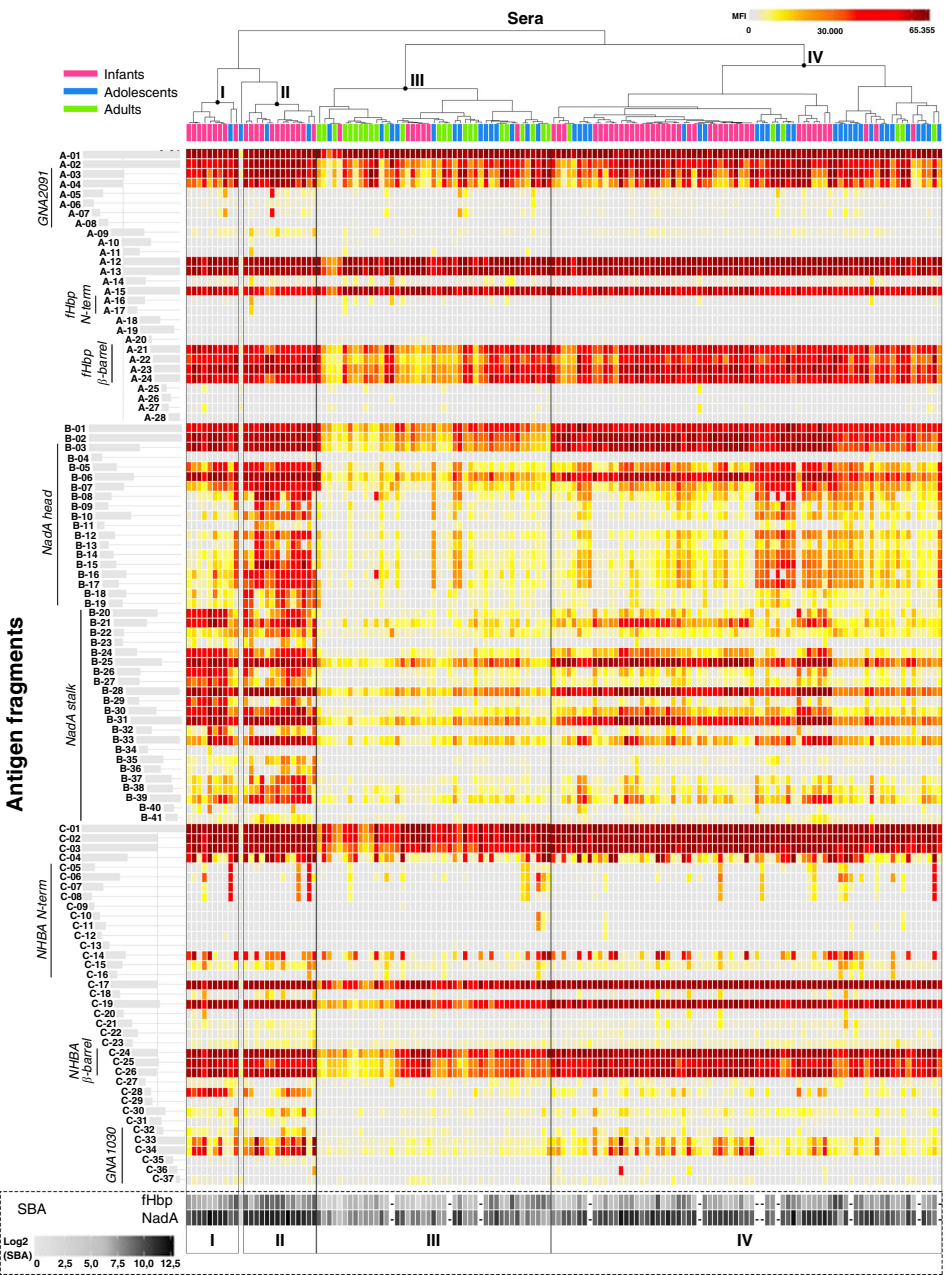
Clustering analysis of protein microarray data and corresponding SBA titres. Clustering analysis of the mean fluorescence intensity (MFI) data obtained from all antigen fragments (rows) tested against single subject sera (columns). Colour scale of signal intensity is reported on top-left of the heatmap. Subjects are coloured according to age group: in green adult subject sera, in blue sera from adolescents, and in pink sera from infants. Rectangles enclose the four major clusters composed of similar sera reactivity profiles. Roman numbers on the top and bottom of the figure identify the four sera clusters considered for subsequent analysis. Individual SBA titres of subjects against fHbp and NadA reference strains are reported below the heatmap according to a grey scale code, SBA data for ten adolescents and one adult were not available as indicated by an ‘*’ symbol in the same box. No NHBA reference strain was available when the studies were conducted.

This microarray analysis revealed the specific recognition patterns of MenB vaccine components targeted by vaccination-induced antibodies of vaccinees in different age groups. To get a broader perspective from single subject recognition profiles, all MFI data underwent hierarchical clustering according to overall antigen fragments reactivity; in Fig. 3, sera with similar recognition profiles for the three vaccine antigens were positioned next to each other. This analysis resulted in a dendrogram that was validated with bootstrap resampling^41^.

This unsupervised hierarchical clustering analysis pointed out four major clusters, which varied in composition and recognition profile (Fig. 3). Clusters I and II were mainly composed of infants that were about 90% of the subjects in both groups, and a number of infants could be found also in cluster IV. Conversely, the majority of adults were grouped together in cluster III. Adolescents were mostly spread between clusters III and IV (Table 1).

**Table 1:** Details of major clusters composition.

Cluster	I	II	III	IV	Total
Infants	9	12	7	41	69
Adolescents	1	2	14 (12§)	28 (20§)	45^a^ (35§)
Adults	0	0	24 (23§)	6	30
Total	10	14	45 (42§)	75 (67§)	144^a^ (134§)

Clusters composition in terms of number of subjects per age group over the total number of subjects per age group.

^a^One adolescent over the total of 46 showed a very peculiar low reactivity and was not classified in any cluster.

The sera response resulted highly variable for NadA antigen with some sera recognising all the spotted specific NadA fragments and some other sera only the full-length proteins. This variability strongly influenced the clustering algorithm and guided the cluster composition. Conversely, we found a quite homogeneous response for fHbp antigen in which most of the human sera displayed a similar recognition profile. Differently, NHBA antigen exhibited substantial variability mainly for the N-terminal region of protein, while the response versus the NHBA β-barrel appeared quite homogenous and abundant.

Besides, the available bactericidal data (Serum Bactericidal Aassay or SBA) against H44/76 and 5/99, indicator stains for fHbp and NadA, respectively^42–46^, were visualised at the bottom of the cluster (Fig. 3) to directly compare the recognition pattern of each serum to its functional property. Bactericidal data on NHBA indicator strain were not available at the time of the clinical studies, therefore NHBA was not considered in this analysis.

**Fig. 4:**
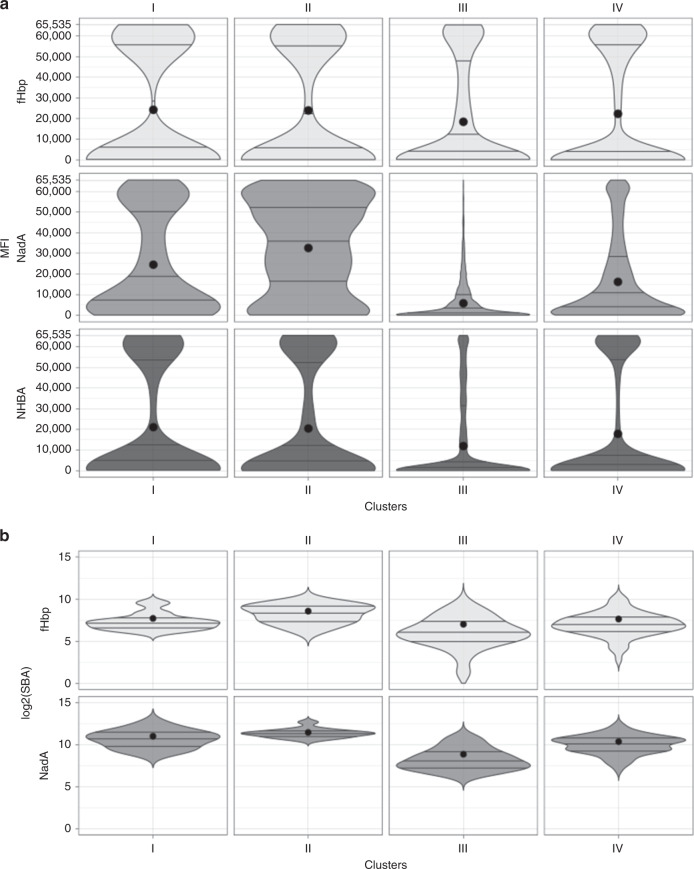
Details of major clusters sera reactivity and functional activity. Panels **a** and **b** respectively represents violin plots of MFIs of all specific antigen fragments and reference strain SBA titres available (vertical axes) in each of the four major clusters (horizontal axis). Black dots represent the mean of the distributions, while horizontal lines indicate quartiles. The wider sections of the violin plots represent a higher density in their data distributions, while the skinnier sections represent a lower density. § since the SBA data were not available for 11 subjects, the panel **b** violin plot for cluster III represents 7 infants, 12 adolescents and 23 adults; the plot for cluster IV represents 41 infants, 20 adolescents and 6 adults.

Specifically, in fHbp cluster analysis nearly all sera reacted predominantly with full-length antigen or fragments covering the whole C-terminal β-barrel, varying only in the intensity level. In particular, the lowest mean MFI value of fHbp specific fragments was observed in cluster III, even though the difference with the other clusters was not statistically significant (Fig. 4a). The recognition of the NadA stalk was less variable if compared to the head domain. As for the fHbp, the greatest mean MFI value of NadA specific fragments was found in cluster II, due to the broad reactivity of all NadA fragments, either as full-length or long or short fragments covering the head or the stalk domain. The mean protein microarray reactivity of the clusters strongly correlated (*R*^2^ = 0.9993; Supplementary Fig. 2) with mean serum bactericidal activity (SBA) titres for 5/99 strain (NadA reference strain), for which we observed the same trend with the highest SBA titre in cluster II (max titre: 2803), and the lowest in cluster III (min titre: 465) (Fig. 4b).

On the other hand, clustering analysis revealed a different response to NHBA among the age groups; in addition, it has also been observed an inter-individual variation, even if less pronounced respect to NadA. As previously mentioned, almost all subjects recognised the β-barrel domain (C-24, C-25 and C-26), while recognition of the N-terminus was more age dependent, in line with phage display result. In particular, 35% of sera showed also antibodies directed versus the central part of the protein (149–275 aa, fragment C-14) and those subjects are prevalently adolescents spread among the four clusters. Moreover, only 14% of sera, exclusively from adult and adolescent subjects, were found highly reactive against fragments covering the unsolved N-terminal portion of NHBA (from C-05 to C-13). Also for this antigen, the lowest reactive sera clustered in group III (Fig. 3).

### Protein array reactivity correlates with bactericidal titre

**Fig. 5:**
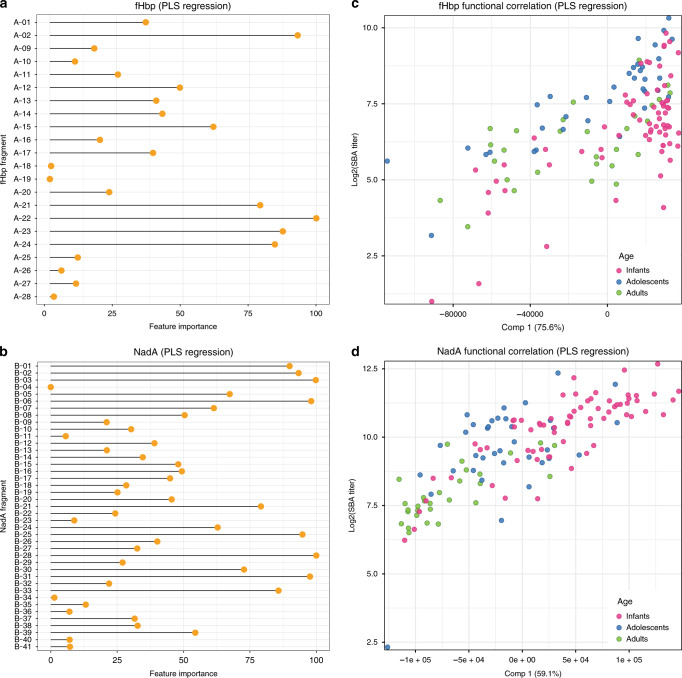
Correlation of bactericidal activity with specific or general protein array reactivity. Panels **a** and **b** respectively represent the single fHbp and NadA fragments contribution to the PLS regression model of the SBA outcome through MFI fragment signals. The fragment importance score corresponds to a weighted sum of the absolute regression coefficients, values > 75% suggest the relevant predictors of the model. Scatterplots of the absolute value of global MFI first PLS component scores against the log2 of SBA titres for respectively fHbp (panel **c**) and NadA (panel **d**) antigens in all age groups. Source data are provided as a Source Data file.

To understand whether there was a significant relationship between the reactivity of sera tested with the protein arrays and the functional SBA data, partial least square (PLS) regression was applied to model the SBA outcome through antigen fragment MFIs signals. For the fHbp antigen, the SBA titres were measured against H44/76 reference strain. For this antigen, PLS modelling selected 13 principal components returning an *R*^2^ = 0.44 and explaining 99.8% of the variation contained in the predictors, and 57.8% of the variation in the outcome variable. Figure 5a shows that SBA titres correlated with the reactivity of mostly fHbp fragments containing the complete β-barrel (A-02, A-21, A-22, A-23, A-24: importance score > 75%). The contribution to the correlation was lower (importance score < 50%) for the full-length antigen (A-01, A-12 and A-13) possibly because the reactivity of these long fragments reached the MFI upper limit of detection, thus truncating their signal to the maximum value (65,535).

In the case of the NadA antigen, the SBA response was tested against the NadA 5/99 reference strain and the PLS modelling selected 5 principal components with an *R*^2^ = 0.63 which resulted in 88.4% variation in the predictors, and 72.1% in the outcome variable. As shown in Fig. 5b, a higher number of NadA fragments, compared to fHbp, resulted to significantly contribute to the correlation with the bactericidal titres. Interestingly, the NadA fragments with the highest contribution to the correlation (score ≥ 75%) were located both at the N-terminal head or C-terminal stalk domains of NadA.

PLS dimensionality reduction allows to compare the whole antigen fingerprinting profile of every single subject serum with the SBA titres measured with the same sera. fHbp first principal PLS component score values (Comp 1, explaining 76% of predictors and 41% of outcome variances) were compared with individual SBA titres of fHbp reference strain H44/76 (log_2_). Figure 5c shows the scatterplot of absolute value of the individual Comp 1 scores against the log_2_ of SBA titres, coloured by age group. Adult and adolescent sera were distributed along a line, while infant sera cluster more at the right side, due to the proximity of the reactivity signals to the MFI upper limit of detection. The same happens for NadA, in which the comparison of the PLS model component (Comp 1, 59% of predictors and 64% of outcome variances) score values with individual SBA titres of NadA reference strain 5/99 resulted in a very high correlation (Fig. 5d).

Overall, the PLS regression suggests that the recognition pattern of the fragments is a good correlate of the functional response (i.e. serum bactericidal activity). In particular, the estimation of the features (fragment related signals for each antigen) that more contribute to the model showed that the reactivity of specific fragments, corresponding to defined protein antigen structural elements, is needed to explain the measured SBA outcome.

## Discussion

The development of new vaccines would be greatly expedited by the use of effective methods to predict vaccine performance. Such methods could also be useful in evaluating individual and age-related responses to existing vaccines. Here, the combined use of phage display technologies and computational approaches led us to build a simple and effective microarray based method, which could be applicable to any protein based vaccine for the dissection of the immune response elicited in individual subjects. In particular, this method allowed a detailed assessment of the antibody responses towards the protein components of the meningococcal vaccine 4CMenB, by comparing immune response against the epitopes of the three recombinant antigens among different age groups of vaccinees. As a result, we detected specific differences in individual antibody response, allowed the definition of an immunosignature that overall correlated with bactericidal activity.

The antibody recognition profile defined by phage display in pools of sera from infants, adolescents and adults was highly positive against the three main 4CMenB antigens after vaccination underlying the good immunogenicity of this vaccine from the infancy to the adulthood. Interestingly, the phage display approach revealed, especially for NHBA, a different profile of response in the three age groups, leading to a broader response against all protein epitopes in adults. The *nhba* gene is ubiquitous in meningococcal strains of all serogroups and it is also found in *N. gonorrhoeae* as well as in different commensal Neisserial species, including *Neisseria lactamica*, *Neisseria polysaccharea*, and *Neisseria flavescens*^47–49^. This age-related effect could be explained considering the presence of pre-existing natural immunity to meningococci^6,50^, or other commensal species whose colonisation incidence rises with the increase in age, and can influence the antibody profile following immunisation.

Because of the difficulties in immunological screening a phage display library, in which DNA from single positive plaques need to be isolated and sequenced, this approach was limited to the screening by using pooled sera.

The identification of the most immunogenic epitopes by phage display allowed the design of an ad hoc protein microarray, in which the overlapping immunoreactive fragments spanning the entire sequences of NadA, NHBA and fHbp were spotted on the chips. With tiny amount of sera (∼1 µl), it was possible to screen multiple sera deriving from immunisation with the 4CMenB vaccine and dissect the immune response in the single subjects in terms of epitope recognition and inter-individual variability. Interestingly, the majority of the sera recognised larger antigens’ domains, respect to short peptides, suggesting that most of the antibodies were directed against conformational epitopes, made up by discontinuous peptides. The availability of epitope recognition patterns obtained from single sera permitted to correlate the immune reactivity to specific protein fragment with the functional activity of the antibodies.

Remarkably, although the two methodological approaches were different, the overall antibody profile against the recombinant antigens was confirmed. In fact, the main recognised regions were the C-terminal β-barrel of NHBA and fHbp, while the response against NadA was directed towards different domains spanning the entire protein. The easiness and the rapidity of the microarray assays allowed to extend the analysis also to single subjects of the infants’ cohort. A different and larger panel of sera was used for the microarray analysis of infants’ and adolescents’ cohorts, to dissect more in detail the immune response in the most relevant target population of 4CMenB vaccination.

One of the major advantages of the protein array has been the possibility of analysing multiple single subject sera to study the correlation between the immune-profile in single subject and the functional bactericidal response. A clear inter-individual variable response against the three antigens was observed, as some sera gave positive signal only with few epitopes while other sera were recognised the majority of fragments. In particular, the immune response against NadA was the most variable, with some subject sera recognising only the stalk region and others recognising the full antigen, independently by the age of the immunised subjects from which the sera were derived. As discussed above, antibody recognition to the N-terminal domain of NHBA was only evident in a small number of adult and adolescent sera. Immune response against fHbp was less variable among individuals and prevalently directed towards the C-terminal β-barrel region. Because of the variability in response in the different subjects and age groups, a clustering analysis was applied to define the major groups with different epitope recognition patterns, and four main clusters were identified. Bactericidal data against NadA and fHbp measured using specific MenB indicator strains and the mean bactericidal titres of individual subjects within each cluster were calculated. Particularly for NadA, a strong positive correlation was observed between the reactivity of each cluster, in terms of mean MFI signals, and the corresponding mean SBA titres. Remarkably the most reactive cluster included sera from infants and adolescents, showing that 4CMenB induces a strong effective response in these age groups, which are more at risk of disease. For NHBA, currently this correlation cannot be evaluated due to the lack of SBA data specific for this antigen; future studies will be dedicated to map its protective and non-protective epitopes.

Overall, our results indicate that the vaccine recombinant antigens contain multiple protective epitopes and that full-length proteins are required for the elicitation of an optimal functional activity. In particular, for NadA, the concomitant presence of antibodies against the N-terminal and the C-terminal regions is responsible for the highly positive SBA titres, suggesting that simultaneous recognition of different antigenic regions by antibodies induced by the vaccine is crucial for functional bactericidal activity (Fig. 5), as was already pointed out by Giuliani et al.^40^.

Important evidence arises from the analysis of the response against fHbp. This antigen exists in three variant groups with limited cross-protective responses. We observed that the immune response against fHbp is prevalently directed towards the C-terminal β-barrel and the fragments were recognised only when they were sufficiently long to encompass the entire domain, suggesting that most of the antibodies elicited by vaccination were directed against conformational epitopes. This finding is in accordance with the characterisation of fHbp immune response with monoclonal antibodies in Giuliani et al.^40^. The C-terminal β-barrel region is the most variable region among the variants of this antigen in circulating strains, and this may explain the poor cross-reactive functional response elicited by each specific variant^51,52^. These results confirm that, although fHbp is a crucial component for vaccine efficacy, the presence of the other vaccine components is very important to increase the coverage of 4CMenB and circumvent the fHbp specificity.

In this study the data on the bactericidal activity induced by 4CMenB vaccination are related to two strains, 5/99 and H44/76, known to be specifically killed by anti-NadA and anti-fHbp antibodies. Therefore, the immune recognition of each antigen was correlated with the bactericidal activity of antibodies targeting one specific antigen on the target strain. However, being the 4CMenB a multicomponent vaccine, additional antigens are known to contribute to bactericidal activity and to the overall strain coverage. Among them, OMV minor antigens are known to play a synergistic role in the cross-bactericidal activity. In a recent study, sera from adults vaccinated with an OMV vaccine were used to screen an antigen microarray containing 91 outer membrane proteins^27^. The authors identified six very high responding antigens corresponding to OMVs components that could play a role in the protective immune response induced by OMV based vaccines. It will be interesting in future to analyse the immune response induced by the 4CMenB vaccination using a protein array tailored on the OMV component of 4CMenB and to fully characterise the contribution of OMV minor antigens to protection.

In this study we showed that using this approach it is possible to characterise the immune response in clinical study samples. In particular, we highlighted how individual immunity and age of recipients influence the recognition pattern of 4CMenB antigens and how this correlate to functional data. This work describes a template procedure that can be applied to hundreds of volunteers in clinical efficacy trials to predict vaccine performance. For many diseases the use of this approach would serve as a discovery tool to rapidly design and develop more effective subunit vaccines.

## Methods

### Human sera from clinical trials

Human serum samples analysed in phage display and protein microarrays derived from three distinct clinical trials. Adults’ sera belong to a phase 2 clinical trial (NCT00560313) evaluating safety and immunogenicity of rMenB+OMV NZ in at-risk (due to occupational exposure to *N. meningitidis*) adults aged 18–40 years according to a 0-, 2- and 6-month immunisation schedule followed at month 7 by a single dose of Menveo (MenACWY conjugate vaccine)^46^. Adolescents’ sera were from a phase 2b/3 study (NCT00661713) conducted in Chile to evaluate different schedules in adolescents 11–17 years of age. Subjects received either one dose, two doses (either 1, 2 or 6 months apart), or three doses of rMenB+OMV NZ^43^. Finally, infants sera derived from an extension study (NCT00944034)^45^ designed to investigate the safety, tolerability and immunogenicity of a fourth (booster) dose of rMenB+OMV NZ at 12, 18 and 24 months of age in subjects previously primed with three doses of rMenB+OMV NZ in the parent study (NCT00721396)^42,53^.

This study used human biological samples from the following sponsored clinical studies, NCT00560313, NCT00661713 and NCT00944034, which were conducted in accordance with the Declaration of Helsinki, International Conference on Harmonisation and Good Clinical Practice.

An informed consent form, which included permission for potential re-use of the biological samples, was obtained for participants in all the clinical trials, with the exception of a subset of participants from a phase 2b/3 study (NCT00661713). Upon discovery of the unintended error, GSK informed the principal investigators, the local Ethics Committee and the Ministry of Health of the concerned country and notified all impacted participants.

For NCT00560313, Ethical committee approval was obtained from Comitato Etico Locale per la Sperimentazione Clinica dei Medicinali (Azienda Ospedaliera Universitaria Senese di Siena, Italy) and Ethik-Kommission der Phillipps(Universität Marburg, Germany).

For NCT00661713, Ethical committee approval was obtained from Comite de Etica en Investigacion en Seres Humanos (Facultad de Medicina, Universidad de Chile, Chile), Comite de Etica de la Investigacion del Servicio de Salud (Metropolitano Norte, Santiago, Chile), Comite de Etica Cientifi co Pediatrico, Servicio de Salud (Metropolitano Oriente, Santiago, Chile), and Comite de Evaluacion Etico Cientifi co del Servicio de Salud (Valparaiso, San Antonio, Chile).

For NCT00944034, Ethical committee approval was obtained from Ethikkommission der LÄK Rheinland-Pfalz (Germany), Commission d’Èthique Biomédicale (Cliniques universitaires Saint-Luc, Belgium) NHS, National Research Ethics Service (NRES Committee South Central-Oxford A Southwest Research Ethics Committee Centre, UK) and Etická komise FN a LF UP Olomouc (Czech Republic), Comitato Etico Fondazione IRCCS Cà Granda (Ospedale Maggiore Policlinico, Italy) and CEIC de la DGSP y CSISP (Spain).

Due to the limited amount available and the restrictions of ICFs the unique biological samples are not available.

### Construction and selection of λ-phage display libraries

Three Lambda phage libraries were individually generated for all three recombinant MenB vaccine antigens: NadA, GNA2091-fHbp and NHBA-GNA1030. The genes encoding for the three MenB vaccine antigens NadA, GNA2091-fHbp and and NHBA-GNA1030 were amplified from expression plasmids used to produce the recombinant antigen (pET-24b+) using the following primers respectively: 961cL forward (5′-AAACACTTTCCATCCAAAGTACTGACCAC-3′) and 961cL reverse (5′-ACCCACGTTGTAAGGTTGGAACAGAC-3′), GNA2091-fHbp_forward 5′-ATGGTCAGCGCAGTAATCGGAA-3′ and GNA2091-fHbp_reverse 5′-TTGCTTGGCGGCAAGGCCGATA-3′, NHBA-NUbp_forward (5′-CCCGATGTTAAATCGGCGGACA-3′) and NHBA-NUbp_reverse (5′-TTGTTTGGCTGCCTCGATTTGGAT-3′). The amplified product was purified and randomly digested with Dnase I and fractionated by 2.5% agarose gel electrophoresis in order to obtain 200–1000 bp fragments. These fragments, ligated with specific adapters, were cloned into the λKM4 phage^16^ as fusions to the N-terminal fusion proteins with gpD, a λ-phage capsid component that is exposed on phage surface. The lambda library was packaged in vitro using the Gigapack III Gold Packaging Extract (Stratagene). All libraries had random distributions of size and sequence of gene fragments and sufficient diversity, as assessed in previous studies by next generation sequencing^32,39,54,55^. The average size of DNA inserts was 330 bp for GNA-fHbp, 370 bp for NadA and 408 bp for NHBA-GNA1030 and the total number of independent clones was 8.6 × 104, 2 × 104 and 6.1 × 104, respectively.

The affinity selection of the display library with antibodies from serum samples, isolation of recombinant phage clones and identification of each phage insert were performed as described below. The libraries were subjected to affinity selection by incubating magnetic beads linked to Protein G (Dynabeads Protein-G; Dynal) with library-serum mixtures for 1 h at RT under agitation. After washing 10 times with 1 ml of washing solution (1× PBS, 1% Triton, 10 mM MgSO_4_), bound phage particles were recovered by infection of LE392 *Escherichia coli* cells added directly to the beads. After a 20-min incubation, 10 ml of molten NZY-top agar (48 °C) was added to the mixture of infected cells and immediately poured onto NZY plates (15 cm). After overnight incubation, the phage particles were harvested by gentle agitation with 15 ml of SM buffer for 4 h at 4 °C. The phage particles were purified by PEG/NaCl precipitation and stored at 1/10 of the initial volume, then used for one subsequent selection round. The libraries did not show any significant reactivity with sera from pre-vaccinated individuals. Recombinant inserts from single clones were analysed by PCR amplification (primers K47 5′-GGGCACTCGACCGGAATTATCG-3′ and K48 5′-GGTATGAGCCGGGTCACTGTTG-3′) and then sequenced (primers C1 5′-CGGAATTATCGATTAACTTTATTATT-3′ and C2 5′-GTTGCCCTGCGGCTGGTAAT-3′). Sequenced data were analysed with Sequencher 5.0 (Gene Codes) and then with a Perl script that gives for each sequence the following information: if the insert sequence is in the correct reading frame, insert length, start and end position on the corresponding protein sequence. Using an R script, it was calculated the occurrence of each amino acid position of reference protein sequence by summing the counts of all inserts in the corresponding position and normalising the results with the number of totals in frame clones obtained by the corresponding selection experiment.

### Protein array design, generation and validation

Fragments were high-throughput cloned on several commercially available vectors using the polymerase incomplete primer extension method^56^ as tag-less (full-length only) or fusion with different purification tags. When predicting epitopes, we avoided the interruption of protein domains using a secondary structure prediction software [https://npsa-prabi.ibcp.fr/cgi-bin/npsa_automat.pl?page=/NPSA/npsa_seccons.html].

Immobilized metal affinity chromatography purified proteins from the bacterial soluble fraction were analysed by sodium dodecyl sulfate polyacrylamide gel electrophoresis and matrix-assisted laser desorption/ionization time-of-flight to confirm their identity and to assess their integrity and purity.

In total, we spotted on the arrays 41 specific NadA full-length or fragments, 37 specific for NHBA-GNA1030 and 28 specific for GNA2091-fHbp (Supplementary Fig. 1). Each protein was spotted in duplicates per array onto ultra-thin nitrocellulose coated glass slides (FAST slides; Maine Manufacturing) using the ink-jet spotter Marathon (Arrayjet) resulting in spots of ~110 μm in diameter. Printing was performed in a cabinet with controlled humidity and temperature (55–60% and 12 °C, respectively)^40^.

Preliminary array validation was carried out to confirm the efficiency and reproducibility of the protein deposition and immobilisation on the chips, some test slides were probed with anti-GST polyclonal antibodies (Ray Biothec, Inc. 168–10761) 1:500 and rabbit anti-His6 tag polyclonal antibodies (Genetex-GTX77352 1:500); followed by detection with an AlexaFluor^®^647-conjugated anti-rabbit IgG secondary antibody (Jackson Immunoresearch 111-605-046) 1:800. To verify protein fragments are properly spotted on the slides, some test slides were probed with a panel of 6 monoclonal antibodies 30G4 and 12C1 anti-fHbp; 33E8, 6E3 and 9F11 anti-NadA and 31E10 anti-NHBA diluted at 0.5 pg/µl followed by detection with an AlexaFluor^®^647-conjugated anti-mouse IgG secondary antibody (Jackson Immunoresearch 115-605-062) diluted 1:800.

For human sample profiles the assay was performed at room temperature and consisted in a two-step immunofluorescent assay. After a saturation step with Block-It (ArrayIt) for 1 h, plasma samples were diluted 1:500 in Block-It and incubated for 1 h prior washing with PBS-Tween 0.1% (PBST) and incubating another hour with AlexaFluor^®^647-conjugated rabbit anti-human IgG (Jackson ImmunoResearch 309-605-008) 1:800. Then, after final washes with PBST, PBS and distilled water, fluorescence signals were detected by using the PowerScanner confocal laser scanner (Tecan Trading AG, Switzerland) and the 16-bit images were generated with the PowerScanner software v1.2 (Tecan Trading AG, Switzerland) at a 10 μm/pixel resolution. Images were processed using the ImaGene 9.0 software (Biodiscovery Inc., CA). Microarray data analysis was performed using in-house developed software and R scripts. For each protein, the MFI of replicated spots was determined after subtraction of the background value surrounding each spot and the MFI of the corresponding tag. Signals were considered as positive when their MFI value was higher than 5000, corresponding to the MFI of protein spots after detection with rabbit AlexaFluor^®^647-labelled anti-human antibody, plus 10 standard deviation values.

### Statistical analysis

Nonparametric two-sided Wilcoxon rank-sum test was carried out in R statistical environment on each full-length antigen and all its fragments MFI to estimate statistically significant differences between the reactivity distributions of the three age groups. *p* Values were computed in R with wilcox.test function (stats v3.1.1) and false-discovery rate was estimated with qvalue (qvalue v1.38.0). True positives were considered when both *p* and *q* values were less than 0.05.

For the unsupervised hierarchical clustering, a nonparametric bootstrap test (sampling with replacement) was performed on sera reactivity profiles (MFI values of every antigen and fragment of all subjects) in R to determine whether the clusters found by an unsupervised method appeared to be robust in the given dataset with BootstrapClusterTest function (ClassDiscovery v3.3.0). Clustering was executed with ‘complete-linkage’ agglomeration method of cutHclust function, ‘Manhattan’ as distance metric, 10,000 repetitions and *k* = 6.

A dendrogram with approximately unbiased (AU) *p* values were built on top of previous bootstrap agreement results with the pvclust function (pvclust v1.3.2), ‘complete-linkage’ method, ‘Manhattan’ algorithm of distance and 1000 repetitions. Clusters with AU values greater than 0.9 were considered statistically significant.

Preimmune sera were excluded from hierarchical clustering due to their consistently negative response. The correlation between antigen fragments MFIs and SBA titres of all subjects was performed in R by PLS regression modelling with train function (caret v6.0). PLS machine learning modelling was selected for its robustness in case of features correlation and low dimensionality datasets and because it is both a technique of dimensionality reduction and a method to identify the relevant fragments contributing to the correlation with SBA titres. The training of the regression model was directed to *R*^2^ correlation parameter maximisation with a simple leave one out cross validation approach. Default grid optimisation on a maximum of 20 components led train function to the selection of the optimal model. Features (fragments) relevance in the model was measured by importance score (corresponding to weighted sums of the absolute regression coefficients) computed with varImp function.

### Reporting summary

Further information on research design is available in the Nature Research Reporting Summary linked to this article.

## Supplementary information

Supplementary Information

Reporting Summary

## Data Availability

Source data are provided with this paper as a Source Data file. The datasets generated by Protein microarray experiments and analysed during the current study are available in the Gene Expression Omnibus database GEO GSE152785. Phage display raw data will be available on request. Source data are provided with this paper.

## Source data

Source Data

## Code avaiability

Custom scripts developed in R and Perl languages are available on the GitHub repository [https://github.com/muzzial1/4CMenB_fingerprinting_Nat_Comm]. Source data are provided with this paper.
