# Supplementary material for: Immunological fingerprint of 4CMenB recombinant antigens via protein microarray reveals key immunosignatures correlating with bactericidal activity
Source: Nat Commun. 2020 Oct 5;11:4994. doi: 10.1038/s41467-020-18791-0 (PMC7536418; doi:10.1038/s41467-020-18791-0)
Supplement: Supplementary file 1 — Supplementary Information [file 41467_2020_18791_MOESM1_ESM.pdf]

Immunological fingerprint of 4CMenB recombinant antigens via protein microarray reveals key immunosignatures correlating with bactericidal activity

E. Bartolini *et al.*

Supplementary Information

Supplementary Figures

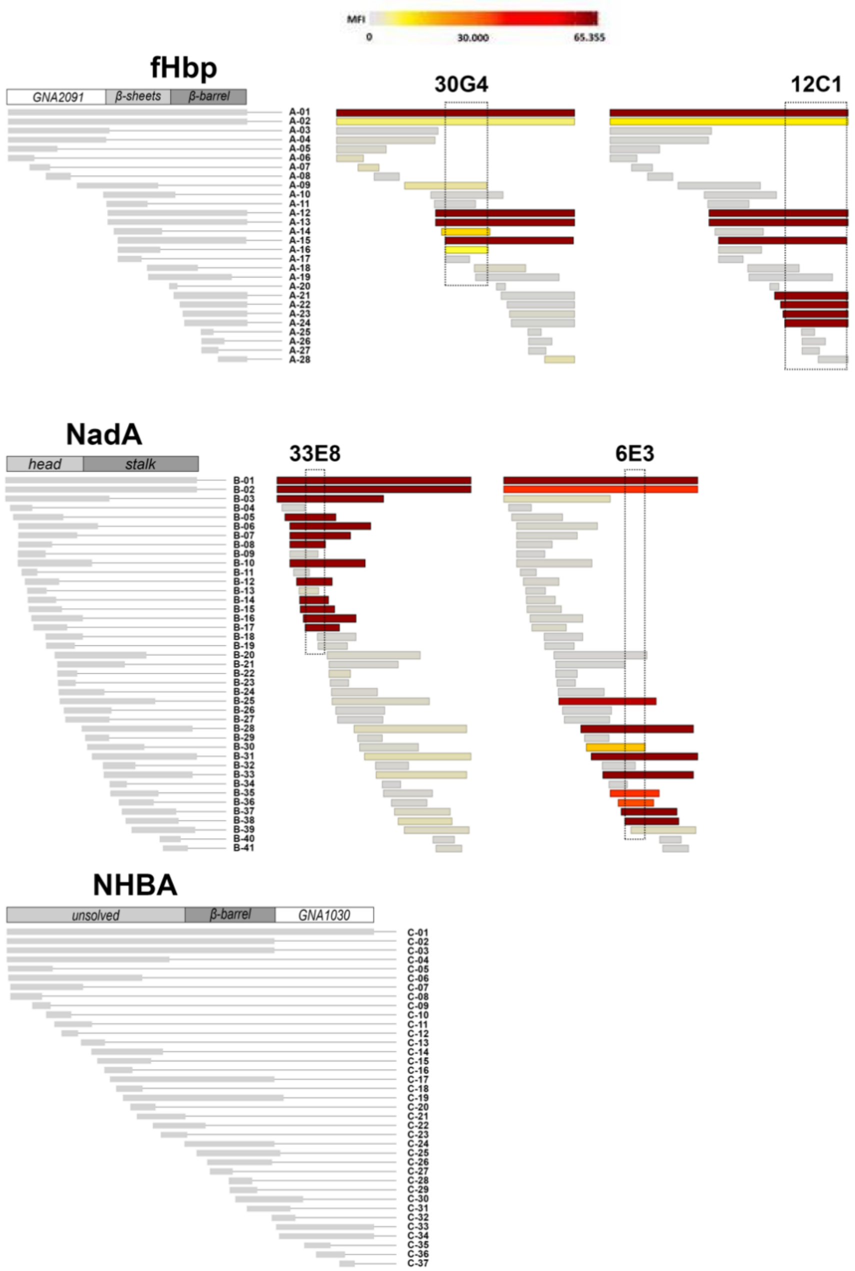

Supplementary Fig. 1. Left: schematic view of all spotted fragments in the array represented as bars, covering the whole sequence of the three antigens: GNA2091-fHbp, NadA and NHBA-GNA1030. Right: epitope mapping analysis of a panel of well-characterized monoclonal antibodies (mAbs). Colour scale reports MFI of every antigen fragment recognized by mAbs. Protein array results for 31E10 anti-NHBA and 9F11-anti NadA mAbs were previously published and reported in Domina *et al.* (*1*). Cariccio *et al.(2)* respectively.

MFI

fHbp

A	B	p.two.tailed	p.greater	p.lesser	Response
1	2	9,60E-01	5,20E-01	4,80E-01	Cluster 1 = Cluster 2
1	3	4,33E-01	2,17E-01	7,84E-01	Cluster 1 = Cluster 3
1	4	5,39E-01	2,69E-01	7,31E-01	Cluster 1 = Cluster 4
2	3	3,73E-01	1,86E-01	8,14E-01	Cluster 2 = Cluster 3
2	4	4,74E-01	2,37E-01	7,63E-01	Cluster 2 = Cluster 4
3	4	5,54E-01	7,23E-01	2,77E-01	Cluster 3 = Cluster 4

NadA

A	B	p.two.tailed	p.greater	p.lesser	Response
1	2	3,47E-06	1,00E+00	1,74E-06	Cluster 1 < Cluster 2
1	3	7,35E-56	3,68E-56	1,00E+00	Cluster 1 > Cluster 3
1	4	1,66E-10	8,30E-11	1,00E+00	Cluster 1 > Cluster 4
2	3	8,20E-121	4,10E-121	1,00E+00	Cluster 2 > Cluster 3
2	4	2,91E-49	1,45E-49	1,00E+00	Cluster 2 > Cluster 4
3	4	1,18E-78	1,00E+00	5,90E-79	Cluster 3 < Cluster 4

NHBA

A	B	p.two.tailed	p.greater	p.lesser	Response
1	2	8,11E-01	4,06E-01	5,95E-01	Cluster 1 = Cluster 2
1	3	1,36E-07	6,79E-08	1,00E+00	Cluster 1 > Cluster 3
1	4	2,69E-02	1,34E-02	9,87E-01	Cluster 1 > Cluster 4
2	3	2,64E-08	1,32E-08	1,00E+00	Cluster 2 > Cluster 3
2	4	2,93E-02	1,46E-02	9,85E-01	Cluster 2 > Cluster 4
3	4	1,20E-06	1,00E+00	6,01E-07	Cluster 3 < Cluster 4

SBA

fHbp

A	B	p.two.tailed	p.greater	p.lesser	Response
1	2	5,59E-02	9,76E-01	2,80E-02	Cluster 1 = Cluster 2
1	3	2,36E-02	1,18E-02	9,89E-01	Cluster 1 > Cluster 3
1	4	5,65E-01	2,82E-01	7,23E-01	Cluster 1 = Cluster 4
2	3	8,28E-05	4,14E-05	1,00E+00	Cluster 2 > Cluster 3
2	4	2,45E-03	1,23E-03	9,99E-01	Cluster 2 > Cluster 4
3	4	4,61E-03	9,98E-01	2,30E-03	Cluster 3 < Cluster 4

NadA

A	B	p.two.tailed	p.greater	p.lesser	Response
1	2	8,41E-02	9,63E-01	4,21E-02	Cluster 1 = Cluster 2
1	3	5,61E-05	2,81E-05	1,00E+00	Cluster 1 > Cluster 3
1	4	8,82E-02	4,41E-02	9,57E-01	Cluster 1 = Cluster 4
2	3	5,32E-08	2,66E-08	1,00E+00	Cluster 2 > Cluster 3
2	4	7,32E-06	3,66E-06	1,00E+00	Cluster 2 > Cluster 4
3	4	3,51E-09	1,00E+00	1,76E-09	Cluster 3 < Cluster 4

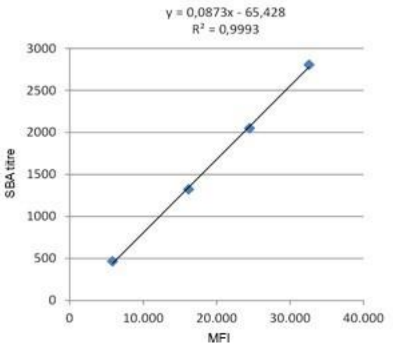

Supplementary Fig. 2. Results of Wilcoxon rank-sum nonparametric test for estimating which clusters showed higher MFI/SBA distributions for the different antigens. Each cluster pair was tested (A vs B) and p-values lesser than 0.05 indicate if overall MFI or SBA distribution of cluster A is significantly greater or lesser than corresponding cluster B distribution. Bottom-right: linear correlation between overall mean clusters MFI on the x axis and mean clusters SBA values on the y axis. R^2 of 0.9993 indicates a very strong correlation.

Supplementary references

1. M. Domina *et al.*, Epitope Mapping of a Monoclonal Antibody Directed against Neisserial Heparin Binding Antigen Using Next Generation Sequencing of Antigen-Specific Libraries. *PLoS One* **11**, e0160702 (2016).
2. V. L. Cariccio *et al.*, Phage display revisited: Epitope mapping of a monoclonal antibody directed against Neisseria meningitidis adhesin A using the PROFILER technology. *MAbs* **8**, 741 (May-Jun, 2016).
