# Supplementary material for: Immunological fingerprint of 4CMenB recombinant antigens via protein microarray reveals key immunosignatures correlating with bactericidal activity
Source: Nat Commun. 2020 Oct 5;11:4994. doi: 10.1038/s41467-020-18791-0 (PMC7536418; doi:10.1038/s41467-020-18791-0)
Supplement: Supplementary file 2 — Reporting Summary [file 41467_2020_18791_MOESM2_ESM.pdf]

nature research

Corresponding author(s): Erika Bartolini and Domenico Maione, NCOMMS-19-35917

Last updated by author(s): Aug 18, 2020

Reporting Summary

Nature Research wishes to improve the reproducibility of the work that we publish. This form provides structure for consistency and transparency in reporting. For further information on Nature Research policies, see our Editorial Policies and the Editorial Policy Checklist.

Statistics

For all statistical analyses, confirm that the following items are present in the figure legend, table legend, main text, or Methods section.

n/a	Confirmed	
[ ]	[x]	The exact sample size (*n*) for each experimental group/condition, given as a discrete number and unit of measurement
[ ]	[x]	A statement on whether measurements were taken from distinct samples or whether the same sample was measured repeatedly
[ ]	[x]	The statistical test(s) used AND whether they are one- or two-sided<br>*Only common tests should be described solely by name; describe more complex techniques in the Methods section.*
[ ]	[x]	A description of all covariates tested
[ ]	[x]	A description of any assumptions or corrections, such as tests of normality and adjustment for multiple comparisons
[ ]	[x]	A full description of the statistical parameters including central tendency (e.g. means) or other basic estimates (e.g. regression coefficient) AND variation (e.g. standard deviation) or associated estimates of uncertainty (e.g. confidence intervals)
[ ]	[x]	For null hypothesis testing, the test statistic (e.g. *F*, *t*, *r*) with confidence intervals, effect sizes, degrees of freedom and *P* value noted<br>*Give P values as exact values whenever suitable.*
[x]	[ ]	For Bayesian analysis, information on the choice of priors and Markov chain Monte Carlo settings
[ ]	[x]	For hierarchical and complex designs, identification of the appropriate level for tests and full reporting of outcomes
[x]	[ ]	Estimates of effect sizes (e.g. Cohen's *d*, Pearson's *r*), indicating how they were calculated

Our web collection on statistics for biologists contains articles on many of the points above.

Software and code

Policy information about availability of computer code

Data collection	A description and the version of all commercial and open source softwares and code libraries used for the data analysis are detailed in the Methods section of the article. Commercial softwares used for data acquisition are the following: PowerScanner software v1.2, ImaGene 9.0 software, Gene Codes software sequencher 5.0
Data analysis	A description and the version of all commercial and open source softwares and code libraries used for the data analysis are detailed in the Methods section of the article. Open source R packages used for data analysis are the following: stats v3.1.1, qvalue v1.38.0, ClassDiscovery v3.3.0, pvclust v1.3.2, caret v6.0. Custom developed scripts were developed in R and Perl languages and, as stated in the manuscript, they are available Custom scripts developed in R and Perl languages are available on the GitHub repository (https://github.com/muzzial1/4CMenB_fingerprinting_Nat_Comm)

For manuscripts utilizing custom algorithms or software that are central to the research but not yet described in published literature, software must be made available to editors and reviewers. We strongly encourage code deposition in a community repository (e.g. GitHub). See the Nature Research guidelines for submitting code & software for further information.

Data

Policy information about availability of data

All manuscripts must include a data availability statement. This statement should provide the following information, where applicable:

- Accession codes, unique identifiers, or web links for publicly available datasets
- A list of figures that have associated raw data
- A description of any restrictions on data availability

The datasets generated by Protein microarray experiments and analysed during the current study are available in the Gene Expression Omnibus database (GEO; http://www.ncbi.nlm.nih.gov/geo/query/acc.cgi) under series accession number GSE152785. As stated in manuscript Phage display raw data will be available on

request.

Field-specific reporting

Please select the one below that is the best fit for your research. If you are not sure, read the appropriate sections before making your selection.

[x] Life sciences   [ ] Behavioural & social sciences   [ ] Ecological, evolutionary & environmental sciences

For a reference copy of the document with all sections, see nature.com/documents/nr-reporting-summary-flat.pdf

Life sciences study design

All studies must disclose on these points even when the disclosure is negative.

Sample size	Data reported in the manuscript came from a panel of available sera derived from 3 different clinical studies (NCT00560313, NCT00661713 and NCT00944034). No sample size calculation was performed in the present paper where we have reported the results of a descriptive analysis performed with the re-use of sera derived from the clinical trials mentioned above
Data exclusions	One adolescent over the total of 46 showed a very peculiar low reactivity when tested on the protein array and was not classified in any cluster therefore was not reported in figure 4.
Replication	Protein chip measurements are made by multiple replicated probes (>6) on the chip, sera are tested singularly. SBA measurements are single experiment results. Phage display data are the result of >10^4 independent clones tested for each antigen. All attempts to replication was succesfull.
Randomization	Data reported in the manuscript came from a panel of available sera derived from 3 different clinical studies where subject allocation was properly randomized in each study (NCT00560313, NCT00661713 and NCT00944034 corresponding respectively to adult, adolescent or infants age groups). No further randomization was performed in the present paper where we have reported the results of a descriptive analysis performed with the re-use of sera derived from the clinical trials mentioned above.
Blinding	Data reported in the manuscript came from a panel of available sera derived from 3 different clinical studies where subject allocation and data were properly blinded in each study (NCT00560313, NCT00661713 and NCT00944034 corresponding respectively to adult, adolescent or infants age groups).<br>A description of the subjects response profile to 4CMenB vaccine treatment by age group was a main objective of this study, for this reason study and arm assignment was disclosed for specific steps of the analysis. In the phage display experiments sera were pooled in the different groups (adult, adolescent or infants) on the basis of the subject age. Protein array experiments and the corresponding data extraction were performed in blinded mode. Protein array experiments data analysis was performed knowing each subject age group assignment (adult, adolescent or infants).

Reporting for specific materials, systems and methods

We require information from authors about some types of materials, experimental systems and methods used in many studies. Here, indicate whether each material, system or method listed is relevant to your study. If you are not sure if a list item applies to your research, read the appropriate section before selecting a response.

Materials & experimental systems

n/a	Involved in the study
[ ]	[x] Antibodies
[x]	[ ] Eukaryotic cell lines
[x]	[ ] Palaeontology and archaeology
[x]	[ ] Animals and other organisms
[ ]	[x] Human research participants
[ ]	[x] Clinical data
[x]	[ ] Dual use research of concern

Methods

n/a	Involved in the study
[x]	[ ] ChIP-seq
[x]	[ ] Flow cytometry
[x]	[ ] MRI-based neuroimaging

Antibodies

Antibodies used	The following polyclonal secondary antibodies were used: AlexaFluor®647-conjugated rabbit anti-human IgG (Jackson ImmunoResearch) 1:800; AlexaFluor®647-conjugated goat anti-mouse IgG secondary antibody (Jackson Immunoresearch 115-605-062) diluted 1:800; AlexaFluor®647-conjugated goat anti-human IgG secondary antibody (Jackson Immunoresearch 309-605-008) diluted 1:800; AlexaFluor®647-conjugated goat anti-rabbit IgG secondary antibody (Jackson Immunoresearch 111-605-046) 1:800. The following polyclonal primary antibodies were used: rabbit anti-GST polyclonal antibodies (Ray Biothec, inc 168-10761) 1:500; rabbit anti-His6 tag polyclonal antibodies (Genetex-GTX77352)1:500;<br>Previously in house developed monoclonal antibodies (conc 0,5mg/ml: 1:1000): 30G4 and 12C1 anti-fHbp ; 33E8, 6E3 and 9F11 anti-NadA and 31E10 anti-NHBA

Validation	Relevant citation for the following primary antibodies:<br>-rabbit -anti-His6 tag polyclonal antibodies (Genetex-GTX77352) Molecular characterisation of ABC-type multidrug efflux systems in Bifidobacterium longum, Anaerobe 2015<br>-rabbit anti-GST polyclonal antibodies (Ray Biothec, inc 168-10761)M. Bombaci et al., Protein array profiling of tic patient sera reveals a broad range and enhanced immune response against Group A Streptococcus antigens. PLoS One 4, e6332 (Jul 22, 2009).<br>30G4 and 12C1 anti-fHbp: E. Malito et al., Defining a protective epitope on factor H binding protein, a key meningococcal virulence factor and vaccine antigen. Proc Natl Acad Sci U S A 110, 3304 (Feb 26, 2013) and A. Faleri et al., Two cross-reactive monoclonal antibodies recognize overlapping epitopes on Neisseria meningitidis factor H binding protein but have different functional properties. FASEB journal: official publication of the Federation of American Societies for Experimental Biology 28, 1644 (Apr, 2014).<br>33E8, 6E3 and 9F11 anti-NadA E. Malito et al., Structure of the meningococcal vaccine antigen NadA and epitope mapping of a bactericidal antibody. Proc Natl Acad Sci U S A 111, 17128 (Dec 02, 2014) and I. Bertoldi et al., Exploiting chimeric human antibodies to characterize a protective epitope of Neisseria adhesin A, one of the Bexsero vaccine components. FASEB journal : official publication of the Federation of American Societies for Experimental Biology, (Aug 24, 2015).<br>31E10 anti-NHBA: M. Domina et al., Epitope Mapping of a Monoclonal Antibody Directed against Neisserial Heparin Binding Antigen Using Next Generation Sequencing of Antigen-Specific Libraries. PLoS One 11, e0160702 (2016)

Human research participants

Policy information about studies involving human research participants

Population characteristics	A panelof human Biospecimens derived from 3 different clinical studies (NCT00560313, NCT00661713 and NCT00944034) were used for the descriptive analysis reported in the manuscript. NCT00560313: Healthy At-risk for meningococcal disease (due to routine occupational exposure to cultured organisms originating from invasive disease isolates) Adults 18-50 Years; NCT00661713: Healthy Adolescents Aged 11-17 Years and NCT00944034: Healthy Infants (12-24 Months). All Tier 1 characteristics of the BRISQ reporting guidelines have been provided in the original Study protocols and the Primary publications.
Recruitment	The recruitments of participants is described in the primary publication 3 different clinical studies (NCT00560313, NCT00661713 and NCT00944034). In this study we used sera derived from a panel of participants. The guidance for sera selection was only linked to sera availability for the phage-display and the protein microrarray experiments
Ethics oversight	NCT00560313: Ethical committee approval was obtained from Comitato Etico Locale per la Sperimentazione Clinica dei Medicinali (Azienda Ospedaliera Universitaria Senese di Siena, Italy) and Ethik-Kommission der Phillipps-(Universität Marburg, Germany)<br>NCT00661713: Ethical committee approval was obtained from Comite de Etica en Investigacion en Seres Humanos (Facultad de Medicina, Universidad de Chile, Chile), Comite de Etica de la Investigacion del Servicio de Salud (Metropolitano Norte, Santiago, Chile), Comite de Etica Cientifi co Pediatrico, Servicio de Salud (Metropolitano Oriente, Santiago, Chile), and Comite de Evaluacion Etico Cientifi co del Servicio de Salud (Valparaiso, San Antonio, Chile).<br>NCT00944034: Ethical committee approval was obtained from Ethikkommission der LÄK Rheinland-Pfalz, Germany, Commission d'Èthique Biomédicale (Cliniques universitaires Saint-Luc, Belgium) and NHS, National Research Ethics Service (NRES Committee South Central-Oxford A Southwest Research Ethics Committee Centre, UK)

Note that full information on the approval of the study protocol must also be provided in the manuscript.

Clinical data

Policy information about clinical studies

All manuscripts should comply with the ICMJEguidelines for publication of clinical research and a completedCONSORT checklist must be included with all submissions.

Clinical trial registration	Data reported in the manuscript came from a panel of available sera derived from 3 different clinical studies. Trial registration numbers are the following: NCT00560313, NCT00661713 and NCT00944034.
Study protocol	Full trial protocol is available at the following Trial registration numbers: NCT00560313, NCT00661713 and NCT00944034
Data collection	In this study we used sera derived from a panel of participants of the 3 different clinical studies cited above. In particular, we have reported the results of a descriptive analysis performed with the re-use of sera derived from the clinical trials mentioned above.<br>Data collection is described in details in the following primary publications:<br>NCT00661713: M. E. Santolaya et al., Immunogenicity and tolerability of a multicomponent meningococcal serogroup B (4CMenB) vaccine in healthy adolescents in Chile: a phase 2b/3 randomised, observer-blind, placebo-controlled study. Lancet 379, 617 (Feb 18, 2012).<br>NCT00560313 A. Kimura, D. Toneatto, A. Kleinschmidt, H. Wang, P. Dull, Immunogenicity and safety of a multicomponent meningococcal serogroup B vaccine and a quadrivalent meningococcal CRM197 conjugate vaccine against serogroups A, C, W-135, and Y in adults who are at increased risk for occupational exposure to meningococcal isolates. Clinical and vaccine immunology : CVI 18, 483 (Mar, 2011).<br>NCT00944034 M. D. Snape et al., Persistence of Bactericidal Antibodies After Infant Serogroup B Meningococcal Immunization and Booster Dose Response at 12, 18 or 24 Months of Age. Pediatr Infect Dis J 35, e113 (Apr, 2016).<br>Time periods and locales:<br>NCT00661713 clinical trial is a randomised, observer-blind, placebo-controlled, study at 12 sites in Santiago and Valparaíso, Chile. Adolescents aged 11–17 years received one, two, or three doses of 4CMenB at 1 month, 2 month, or 6 month intervals. FSFV

05Jun2008, LSLV 16Dec2010, Report Complete (CSR) 27Jul2011

NCT00560313 randomized clinical trial: Italian and German laboratory staff who work with meningococcal isolates are at increased risk for developing invasive disease relative to the general population. FSFV 20Jul2007, LSLV 09Nov2009, Report Complete (CSR) 04May2010

NCT00944034 clinical trial: An open-labeled, randomised, multicenter phase-2b follow-on European study conducted from 2009 to 2012. FSFV 13Jul2009, LSLV 09Jan2012, Report Complete (CSR) 21Nov2012

Outcomes

Here above and in the manuscript we provided publications relating to the primary outcomes of all clinical trials. We confirm that no secondary nor exploratory outcomes of these clinical trials are reported in the manuscript. In the present paper we have reported the results of a descriptive analysis performed with the re-use of sera derived from the clinical trials mentioned above.
